# Selections in Pathology and Surgery; or, an Exposition of the Nature and Treatment of Local Disease; Exhibiting New Pathological Views, and Pointing out an Important Practical Improvement. Illustrated by Cases

**Published:** 1839

**Authors:** 


### Selections in Pathology and Surgery; or, an Exposition of the Nature and Treatment of Local Disease; exhibiting New Pathological Views, and pointing out an important Practical Improvement. Illustrated by Cases. By *John Davies,* Surgeon to the General Infirmary at Hertford, and late Editor of the London Medical and Surgical Journal. London. Longman and Co. 1839.

Mr. Davies informs the readers of his work, in an Advertisement, that:—

"The Physiological Principles upon which the Pathology of Inflammation here set forth is based, were published eleven years ago, in a Series of Essays, in the *Medical Repository,* and the *London Medical and Surgical Journal,* of which the Author was then Editor. The Principles developed in those Essays are founded upon a long course of Experiments, repeated so frequently as to leave little doubt as to their correctness.

The object of the Second Part of the Work is to bring into general notice a Remedy whose superior Curative Properties, as an external application, appears to be but little known to the Profession. This Remedy is Iodine in a liquid form. Respecting the employment of Iodine as an external remedial agent, the Author lays no claim to originality; but, with the exception of a small volume, published some years ago, by Mr. Buchanan, he is not aware that the mode here recommended for its application has been pointed out to the public. During the last ten years the Author has employed the Remedy in question very extensively, both in Infirmary and private practice, and the object of the present Work is to acquaint the Profession with the result of his experience respecting its use during that period." *Preface.*

It would occupy us too long to enter on the examination of the physiological portion of the volume. We will only say of it that it bears sufficient evidence of the ingenuity and acquirements of Mr. Davies. We shall turn at once to the practical part, which sets forth the powers and the application of iodine, that remedy which bids fair to rival in use, if not in utility, mercury itself.

Mr. Davies states that iodine may be applied with much benefit to diseases and local injuries in which it has hitherto been very limitedly, if at all, employed. The various local affections in which he has used it during the last eleven years, both in hospital and private practice, induce him to set it forth as by far the most efficient topical application in our possession. The mode in which he has generally employed it, has been that recommended by Mr. Buchanan, namely, in the form of tincture; in which form, *certainly,* it possesses remedial properties which it does not exhibit in any other. He goes on to remark:—

"It is not the intention here to treat of iodine as an internal remedy, but we may be permitted, in passing, to observe that we have never witnessed it to produce the effects attributed to it on the testes and mammæ, namely, causing the absorption of these glands. We have administered it internally very extensively, and, in many cases, for several months together, yet *not in one instance* did it reduce the size of the glands already mentioned. We may also be allowed to state that we have been much disappointed in it as an internal remedy, more especially in scrofulous affections, for which it has been cried up as almost a specific. Its curative effects in bronchocele are so well established as to render it unnecessary to do more than mention the fact; but with the exception of that affection, its medicinal virtues, according to our experience, are rather limited, when internally administered. It is, however, proper to mention, that we have found it one of the most useful remedies in cases of long-continued dyspepsia, and in some other affections of the mucous membrane of the digestive canal." 81.

On one point, at all events, we agree with Mr. Davies—the exaggerated virtues attributed to iodine in scrofulous affections. There cannot be a greater delusion than to regard iodine as a sort of specific in scrofula—there cannot be much worse or more mischievous practice than to give it indiscriminately, or even extensively in that composite disease. But we think that Mr. Davies robs iodine of much that is justly due to it, when he almost limits its benefits to dyspepsia. To exclude it from the treatment of the cachectic symptoms that follow syphilis, or the abuse of mercury in certain constitutions, would be doing an injustice to it and to the patient.

It is as an external application that Mr. Davies mainly leans on iodine. And he leans on it too with all his weight. He assures us with confidence that it is very easy of application, mild in its effects on the nerves of the part with which it comes in contact, and capable of being modified in the degree of its strength, so as to suit it to the intensity of the disease and the natural quality of the skin of the patient.

The following are the forms in which Mr. Davies recommends and employs this medicine.

"Both at the infirmary and in private practice we are in the habit of using the iodine in two forms, but by far more frequently in that of tincture in alcohol. The tincture is made by dissolving forty grains of iodine in an ounce of rectified spirit; and its strength is afterwards reduced, when necessary, by the addition of more spirit. The other form is an ioduretted solution, which is made by dissolving thirty-two grains of iodide of potassium (hydriodate of potass) in an ounce of distilled water, and by adding to the solution eight grains of iodine. With this ioduretted solution a lotion is made by the addition of distilled or of common water, varying in strength according to circumstances, from one-eighth to one-fourth of the former.

The strength of the remedy must be determined by the quality of the skin, in the first place. When the skin is thin and delicate in texture, the tincture should be reduced to half its strength, otherwise the cuticle may be raised in blisters, which should always be avoided if possible. When, on the other hand, the cuticle is thick, and of a coarser texture, the tincture may be applied undiluted. In the second place, the strength must be regulated by the nature and intensity of the disease. If, for instance, we have an acute inflammation of any part, and if, most particularly, it shows a disposition to slough, the remedy must be used in its full strength. In all cases of sloughing ulcers; in all cases of irritable ulcers; in all cases of lacerated, contused, or punctured wounds, the strength of the tincture should *not* be reduced—it should be applied undiluted in the first instance; but when the slough has separated, or the irritability has ceased, and when the remaining object is merely to promote the growth of granulations, then the strength ought to be reduced about one-half. In indurations of the glands; in chronic affections of the joints; in inflamed breast; in fact, in all affections whose seat is *under* the substance of the skin, the tincture should be employed in its full strength, unless the skin covering the seat of disease be very delicate or irritable—we mean *naturally* irritable, for the remedy itself would soon deprive it of any *morbid* irritability." 83.

Such are the forms of exhibition; we turn now to the mode.

Suppose, says Mr. Davies, we are called to a case of severe inflammation of the leg, in a stout, robust person: the limb is intensely red, hot, swollen, and glossy, all the way from the toes to above the knee: it is double the size of the corresponding one; and so painful as to disturb the general health—as to cause quickness of the pulse, white tongue, thirst, &c. We immediately *paint* the whole limb with the tincture of its full strength, extending its application from the toes to several inches above the upper margin of the inflammation: the remedy is applied with a camel's hair brush. This is all the local application requisite for the present. The limb is directed to be kept in a horizontal posture, and either to be very lightly covered over with a sheet—which must not come in contact with the skin—or else to be left exposed, according to the temperature of the apartment. In less than twenty-four hours—in less than twelve hours—the swelling will be found to have diminished. At the end of twenty-four hours the skin will be seen much corrugated, showing its contents to have become less in bulk, and the circumference of the limb will measure some inches less than the day before. The diminution will be found to have taken place more particularly towards the upper part of the swelling. We now repeat the application of the tincture, of the same strength. In another twenty-four hours the reduction of the swelling will have gone on rapidly, and only a remnant of the disease will be found to exist. The strength of the tincture must be now reduced to one-half, and its application continued daily, or less often, according to circumstances, until the limb is well. After the second or third applications of the tincture, we sometimes brush the limb over with spirit of wine alone, so as merely to dissolve the iodine which remains on the surface of the skin.

Such, Mr. Davies assures us, are the usual effects of the application in cases of pure phlegmonous inflammation. He will not allow of any further variations than the secondary ones of *degree* of *curative* influence. In every case he promises benefit. Nay, he has never seen more than trivial inconvenience from it. Now and then it has caused a slight watery eruption on the surface of the skin, and a degree of itching in the part, when employed too strong, or when persevered in too long. These eruptions have invariably given way in a few days to a simple evaporating lotion of spirit and water.

The direct effects upon the skin are alluded to. Our readers should be informed, as Mr. Davies will inform them, that the application of the tincture is almost always followed by a desquamation of the cuticle, leaving the part covered by a new, clean, healthy layer of cuticle. This takes place when it has been applied to a healthy part. On the healthy skin it generally causes a sensation of heat, and some degree of smarting after a few minutes; but frequently no smarting or pain is felt—nothing but a slight and agreeable warmth. When applied to a part undergoing the process of inflammation, the pain, which forms one of the essential characters of the disease, soon becomes deadened, and is succeeded by a sensation of warmth, which, in a few hours, ceases also, and leaves the patient free from any sense of pain or heat in the seat of disorder. If applied to the surface of a sloughing ulcer, or to a part on the verge of gangrene, or to a foul irritable ulcer, it is seldom felt by the patient. On a healthy ulcer it produces, generally, a very sharp, smarting pain for a minute or two, but the pain almost immediately ceases and leaves the part in a comfortable state. The same sensation is produced by it when applied to the surface of a recent lacerated wound, or to any healthy raw surface.

Mr. Davies cautions us that, if any itching, or a slight blush of the skin, should follow the repeated applications of the remedy, especially on the lower extremities, it should be discontinued, otherwise the cuticle will rise into small watery pimples, and will tease the patient for a few days; but this effect is produced by it on the *healthy* skin only—when applied, for instance, to the skin covering a diseased joint, or when any induration or swelling is situated under the skin—for Mr. D. has never found it to blister the cuticle and to cause exsudation of lymph from the surface of an *inflamed* skin.

" Neither the ioduretted solution, nor the tincture of iodine as directed to be prepared by the London Pharmacopœia, is applicable to cases of inflamed or of ulcerated skin, for they both contain hydriodate of potass. In either of these forms the Iodine is far preferable for *internal* use, to being dissolved alone in alcohol; for in the latter form it nearly all precipitates when water is added to it, whereas the hydriodate in combination with it renders it perfectly soluble in water or watery infusions. As an external application, however, the hydriodate renders it acrid, and gives it a tendency to inflame the skin and to cause a considerable degree of smarting when brought into contact with any ulcer or ulcerated surfaces. The ioduretted hydriodate lotion is only applicable to affections whose seat is under the skin, such as diseases of the joints, scrofulous tumours, glandular indurations, &c.; but even in most of such cases, the tincture is far preferable, when the patient is so situated as to enable the practitioner himself to apply it as often as necessary. We state this proviso, because we never trust the employment of the tincture to the patient." 85.

The ioduretted hydriodate solution, he goes on to state, like the tincture, produces a desquamation of the cuticle if used strong, but this only becomes observable some time after the lotion has been left off, and the skin has become dry. Mr. D., however, seldom uses this preparation, except in cases where it is not convenient to see the patients so often as required for the application of the tincture, because the latter, if indiscreetly employed, is likely to cause an unpleasant sensation in the skin of the part to which it has been applied. The solution forms a very ready mode for an iodine gargle, in cases of ulcerated sore throat. Diluted with from seven to ten parts of water, with the addition of honey, it forms one of the best kinds of applications of that description. But even in affections of the mouth and throat the tincture is preferable—it is less nauseous to the patient, and quicker and more efficacious in its curative effects.

Mr. Davies proceeds to the consideration of the diseases, for which iodine is beneficial. Erysipelas, Phlegmon, Extensive Sloughing of the Cellular Membrane, Acute Inflammation of the Joints, Inflammation of the Breast, Gout, Enlargement of the Joints, Inflammation of the Absorbents, Carbuncle, Lupus, or Noli me Tangere, Malignant Ulcers of the Tongue and Tonsils, Scrofulous Swelling of the Glands, Whitlow, Chilblains, Lacerated, Contused, and Punctured Wounds, Burns and Scalds, Ulcers, constitute the ample catalogue. We shall pause at some of these complaints, for the purpose of selecting an observation or two upon their management.

*Erysipelas.*—The topical remedies, writes Mr. Davies, commonly used in erysipelas are local bleeding, by leeches; simple evaporating lotions, or lotions medicated with lead, zinc, acetate of ammonia, opium, camphor, or any other ingredients, according to the views or fancies of the practitioner; fomentations, generally of chamomile and poppy heads; poultices, simple or medicated; incisions through the seat of inflammation, as recommended by Mr. Lawrence: scarifications, as recommended by Dr. Dobson: the application of lunar caustic, as recommended by Mr. Higginbotham. Of the *modus operandi* of all these plans, we have already spoken, and the degree of reliance to be placed on each or all of them in erysipelas, is so well known as to render it unnecessary to treat of them specially here. But it will be found, upon trial, that the tincture of Iodine is a topical remedy far more efficacious than any of them, or of all of them put together.

We should remark that Mr. Davies does not speak of erysipelas in a restricted sense. He lauds the iodine not only in the cutaneous, but in the phlegmonoid erysipelas too, and incisions would seem to be regarded by him as quite secondary to it in utility. Of course we cannot pronounce with the authority of experience on this point, not having employed the iodine in such cases. But we confess, and Mr. Davies will pardon the scepticism, that we doubt if iodine or any application can effect what incisions do. In cutaneous erysipelas, the complaint, for the most part, runs a pretty regular course, and, perhaps, external remedies receive more credit than they merit. In the erysipelas phlegmonodes, the skin dies from the distention of the cellular membrane, and our imagination scarcely stretches to the powers of iodine in remedying this.

*Phlegmon.*—Mr. Davies observes that, in what manner the influence of the tincture reaches the vessels, in deep-seated inflammation of the cellular membranes, he does not profess to be able to explain, but that the remedy *does* exert an influence upon them is perfectly certain. In cases where pain and throbbing only exist, and where the inflammation has not yet made its appearance on the skin, it will generally be found that a single application of the tincture, in its full strength, and thickly painted over the seat where the pain is felt, will at once check or cut short the disease. A sensation of warmth, and probably some degree of smarting, will be felt in a few minutes in the part to which the remedy has been applied: this may continue for an hour or two; and when it ceases, the original pain also will generally be found to have ceased. However, it is safer to repeat the application, though no more pain or throbbing be felt; for no harm can arise from so doing.

*Inflammation of the Joints.*—Mr. Davies affirms that the tincture of iodine is of more service here, than any of the remedies in common use, that is, than cupping or leeching, evaporating lotions, fomentations or poultices. But if the tincture be used too strong at first, or applied too frequently, it may give rise to inflammation of the integuments, and cause, or add to the, puffiness of the soft parts external to the joint. The texture of the patient's skin must serve to guide the practitioner, in a great measure, respecting the strength of the tincture and the frequency of its application. It should be at first diluted to about half its strength, or more, if the skin be of a very delicate texture; and, if required, its strength may be gradually increased according to its effects.

"The tincture, diluted, may be applied at once all over the inflamed joint, with perfect confidence that not only no *mischief*, but that *good*, will be the result. But when the disease has been pretty far advanced, and where the swelling has been considerable, we have generally preferred leeching the joint first, and then, a few hours after the bleeding has ceased, to apply the tincture. Whether by getting into the leech-bites the remedy exerts a greater influence on the internal vessels of the joint, we do not profess to know, but the fact is that the application of leeches, in this species of inflammation, previous to the employment of the tincture, tends greatly to assist the good effects of the latter. The remedy will generally require to be applied every day, for two or three times; then every other, or every third day, according to circumstances; the practitioner exercising his discretion according to the condition of the part, and the effect of each application. If, in the intermediate time of the applications, the part should acquire an increase of temperature—as it sometimes does very suddenly, without any evident cause—it will be useful to lay over it a layer of rag soaked in spirit of wine, or in a simple spirit and water lotion. This application will not interfere with the repetition of the iodine. But no lead or zinc lotion, or one medicated in any way, should be employed." 106.

Turning aside from *inflammation* of the *mamma*, in which iodine either prevents suppuration altogether, or renders its amount trivial, we are delighted to perceive that its terrors have been stripped from—

*Gout.*—The curative effects, protests Mr. D. of the tincture of iodine in the pain and inflammation of gout can be appreciated by those only who have witnessed them, or those who have received the benefit of the remedy. He has generally found one or two applications remove the pain almost entirely. He usually dilutes the tincture to about two-thirds its full strength; and if the part affected be the foot, he paints it and the toes well over, all the way from the ankle; or from higher up the leg, if the ankle itself suffers from the disease. The application is repeated next day if any pain, redness, or swelling remains. In fact the disease is treated like common phlegmonous inflammation. When the malady has fixed itself in the fingers and hand, the treatment is the same: the whole hand, fingers, and wrist, are brushed over, and the application is repeated as long as any trace of the disease remains.

Mr. Davies adds, that in very many cases of anomalous pains of the joints, supposed to be gouty or rheumatic, unattended by inflammation, the effect of the tincture of iodine has been very noted. In such cases it ought generally to be used in full strength, unless the texture of the skin be delicate. It first causes a sensation of warmth, amounting in most instances to smarting; and it commonly follows that, when that sensation has ceased, the original pain is no longer felt; or, if felt, it is in a much lower degree than before the application of the tincture.

None but those who have suffered from gout or gouty pains, or those who have had to treat them, can feel due gratitude for the benefits which Mr. Davies promises. He will pity, we are sure, our doubts, because they spring from inexperience of the remedy. We hope to be convinced.

If we hesitate to insert what Mr. Davies says on the management of lupus, it is because the two cases of cure that he relates appear to us *not* to have been instances of the disease. The single case of undoubted lupus, was, unhappily, not benefited.

*Malignant Ulcers of the Tongue and Tonsils.*—If we quote the following passage, it is rather for the purpose of exhibiting our author's confidence, than our own.

"Ulcers of a very troublesome kind often form within the cavity of the mouth. These are occasionally tainted with syphilis, especially when they attack the tonsils, and velum of the palate. Frequently, however, even these parts become the seats of destructive ulcerations, in the production of which syphilis has had no share. We were greatly puzzled respecting the treatment of affections of these seats before we began to employ the iodine; but since then the treatment has been easy enough, and almost uniform in its success—indeed, we may say quite uniform, as far as our experience goes." 115.

*Whitlow*, even, bows to the iodine. Mr. Davies instructs us that, as soon as the throbbing and pain of whitlow are felt to be coming on, the whole finger or thumb (whichever it may be), ought immediately to be painted over with the tincture of iodine, of its full strength. In about twelve hours, the application should be repeated, unless the morbid sensation has ceased, which is often the case, even after the first application. In every case, however, it will be prudent to repeat the remedy two or three times, at intervals of twenty-four hours; except where it is certain that suppuration has actually commenced. He does not remember an instance in which the tincture has failed to subdue the disease. Whenever it is suspected that matter exists, or even when that is doubtful, if the inflammation does not give way in a few hours, a *free* incision should be made into the part: the tincture should then be applied over the finger, as well as over the hand, if at all swelled; and it should be made to insinuate itself freely into the incision. After an hour or two have been allowed to elapse, a small poultice should be applied to the end of the finger—confining it to as small an extent beyond the incision as possible, its object being merely to keep the wound from healing. The same treatment should be repeated daily as long as any vestige of the disease remains.

We *skip* Chilblains, Wounds, Burns and Scalds, and rest for the last time on—

*Ulcers.*—Speaking of specific sores, Mr. Davies observes:—

"We have also used it in several cases of chancre, and have found the ulcer to heal much quicker than under the usual mercurial application, or the nitrate of silver. The tincture has been likewise used in ulcers of a carcinomatous character; and although we should not be justified in speaking of it in positive terms as being capable of curing cancer—certainly not cancer of the mamma—yet so many cases distinguished by scirrhous indurations, accompanied by malignant ulcerations, especially of the lips, tongue, and tonsils, have given way under its use, that, certainly, no topical remedy at present in the possession of surgery seems to equal it in efficacy in affections of that description." 127.

In all cases of sloughing, or irritable, or spreading ulcers of the non-specific sort, the tincture, of full strength, is freely applied by Mr. Davies, over their surface, and to the skin, to the extent of two or three inches round them. Having been allowed to remain for some time, the ulcer is covered over with simple ointment on lint, or with a poultice, the former being generally preferred. The same application is repeated daily until the ulcer becomes clean and healthy. The tincture is then weakened, and the granulations are touched with it every two or three days. Under this plan the cavity of the ulcer fills up rapidly with healthy granulations. Where the ulcer is in a situation to have pressure applied, either a bandage or strapping with sticking-plaster is had recourse to as soon as the surface has become clean and has put on a healthy appearance.

We believe we have abstracted the main practical facts or directions from this volume. It would not be an act of honesty towards the public did we profess to subscribe to all that we have quoted. But neither would it be an act of honesty towards Mr. Davies, to profess that we have given his plan an actual trial. We are aware that in medicine there is no standard to which all disputable points of practice are referrible. We are none of those who bawl incessantly for "principles," meaning by principles their own opinions. Nor are we bigots to the wisdom of our ancestors, a convenient mode of wrapping up laziness in prejudice, and prejudice in a pseudo-respect for truth. But the danger of the present times is the opposite extreme. The most stable opinions, the best established generalizations, are set aside like an old almanac, and each man ventures without a qualm his argosy of theory or of empiricism on the boundless sea of novelty.

To return to the occasion of these remarks—the work of Mr. Davies—we do confess our scepticism of much of the power attributed by him to iodine. But we dare say, that its use as an external application is, in common hands, too restricted; and, at all events, Mr. Davies's statements may reasonably lead to experiments on the part of other surgeons. *They* must recollect that preconceived opinions in regard to the operations of remedies, however well founded they may *seem*, occasionally prove erroneous—that all we know, and all we ever shall know, of the effects of medicines, flows from empirical observation only—and, finally, that no patient experimenter, however much he may mistake in some particulars, fails to correct some misconceptions, and to add some truths to the existing stock.

